# Case Report: Clinical response to anaplastic lymphoma kinase inhibitor-based targeted therapy in uterine inflammatory myofibroblastic tumor harboring *ALK-IGFBP5* fusion

**DOI:** 10.3389/fonc.2023.1147974

**Published:** 2023-03-23

**Authors:** Ting Zhao, Xiaowei Zhang, Xin Liu, Min Ren, Yufan Cheng, Jian Wang, Zhiguo Luo

**Affiliations:** ^1^ Department of Gastrointestinal Medical Oncology, Fudan University Shanghai Cancer Center, Shanghai, China; ^2^ Department of Oncology, Shanghai Medical College, Fudan University, Shanghai, China; ^3^ Department of Head & Neck Tumors and Neuroendocrine Tumors, Fudan University Shanghai Cancer Center, Shanghai, China; ^4^ Department of Pathology, Fudan University Shanghai Cancer Center, Shanghai, China

**Keywords:** uterine inflammatory myofibroblastic, inflammatory myofibroblastic tumor, ALK-IGFBP5 fusion, crizotinib, neutrophil-to-lymphocyte ratio (NLR)

## Abstract

**Background:**

An inflammatory myofibroblastic tumor (IMT) is a mesenchymal tumor with a prevalence ranging from 0.04% to 0.7% worldwide, in which the lung is the most common predilection site, accounting for 33% of cases, followed by the abdomen, pelvis, mesentery, and uterus. Approximately 50% of uterine IMTs present as anaplastic lymphoma kinase (ALK) positive along with *ALK* gene fusion, which lays a solid foundation for the development of ALK-based target therapy to optimize treatment strategies.

**Case presentation:**

Herein we describe a 57-year-old woman who presented with a slow-growing mass in the uterus for over 10 years and then received surgical resection because of significant progressive enlargement of the mass during follow-up. She was diagnosed with uterine leiomyosarcoma (LMS) with no further interventions until recurrence. We revised the diagnosis to uterine IMT based on diffuse ALK expression, *ALK-IGFBP5* gene fusion, and the morphologic features of the tumors by pathology consultation. Based on these, we recommended an ALK tyrosine kinase inhibitor (TKI) treatment, crizotinib (250 mg bid), and she achieved a complete response (CR) with at least 18 months of progression-free survival (PFS). We monitored the dynamics of target lesions and peripheral blood cells at regular intervals through CT scans and routine blood tests during the treatment process. We present patient responses to ALK inhibitor-based targeted therapy with uterine IMT harboring *ALK-IGFBP5* fusion, and the neutrophil-to-lymphocyte ratio (NLR) may be an effective indicator to predict prognosis.

## Introduction

An inflammatory myofibroblastic tumor (IMT) is a distinctive mesenchymal tumor with a prevalence ranging from 0.04% to 0.7% worldwide, in which the lung is the most common predilection site, accounting for 33% of cases, followed by the abdomen, pelvis, mesentery, and uterus (1). Symptoms and signs vary depending on the site of the tumor, while patients with uterine IMT commonly present with pain, tenderness, and abnormal vaginal bleeding (2). The recurrence rate of uterine IMT is approximately 25%, even though surgery has long been recognized as the preferred treatment (3), which lays a solid foundation for the development of chemotherapy and target therapy to optimize treatment strategies.

While considered rare, uterine IMT is diagnosed pathologically according to the criteria established by the World Health Organization (WHO) (4). Approximately 50% of uterine IMTs are driven by the rearrangements of the anaplastic lymphoma kinase (*ALK*) locus on chromosome 2p23 (5), which then results in the dysregulation of ALK expression. Structural rearrangements commonly lead to the expression and activation of ALK, which then create considerable opportunities for chimeric fusion. ALK has now been accepted as a specific diagnostic marker and driver gene for IMT of the uterus (6), and several *ALK* fusion partners have been identified retrospectively, for instance, *FN1*, *DES*, *DCTN1*, *TNS1*, *IGFBP5*, *TIMP3*, *TNC*, *TPM3*, *THBS1*, and *SEC31* (3, 7–10). *ALK* fusion has been confirmed as a therapeutic target for patients with ALK-positive tumors, such as non-small cell lung cancer (NSCLC) (11). Previous studies uncovered patients with uterine IMT harboring *DCTN1-ALK* fusion who achieved an ongoing partial response (PR) with an ALK inhibitor (crizotinib/Xalkori^®^) and a multikinase VEGF inhibitor (pazopanib/Votrient^®^) treatment by Response Evaluation Criteria in Solid Tumors (RECIST) 1.1 criteria (8). However, new therapeutic approaches based on other *ALK* fusion partners are rarely reported and need further exploration.

Here, we describe the case of a 57-year-old woman diagnosed with ALK-positive uterine IMT. Next-generation sequencing (NGS) identified the presence of an *ALK-IGFBP5* fusion in her tumor. She, therefore, received an ALK tyrosine kinase inhibitor (TKI) treatment, crizotinib (250 mg bid), with significant symptomatic improvement and a rapid decrease in her tumor burden. This case reports for the first time that TKI is the preferred treatment option for patients with uterine IMT harboring *ALK-IGFBP5* fusion. Our data not only offer insight into the diagnosis and treatment of uterine IMT but also provide a rationale for patient management.

## Methods

### Patient and treatment

One female patient with uterine IMT was admitted to the Fudan University Shanghai Cancer Center and was treated with an ALK inhibitor, crizotinib, 250 mg bid orally, for a 30-day cycle at Fudan University Shanghai Cancer Center. She provided signed informed consent prior to treatment.

### Histological analysis

Surgical resection specimens of uterine IMT were fixed in 10% formalin, embedded in paraffin, and cut into 5-μm-thick sections. Sectioned tissues were then performed for hematoxylin and eosin (H&E) staining and immunohistochemistry (IHC) staining with ALK (Cat. # 3633S, Cell Signaling Technology, Danvers, MA, USA), AE1/3 (Cat. # 67306S, Cell Signaling Technology), PR (Cat. # 8757S, Cell Signaling Technology), and caldesmon (Cat. # ab183339, Abcam, Cambridge, UK).

### Next-generation sequencing

An NGS, i.e., massively parallel sequencing, of 630 genes was performed to detect the gene expression profile of the tumor sample, in addition to ALK rearrangement and gene fusion type within the tumor. NGS was performed to an average depth of >5,000× on the NovaSeq 6000 platform (Illumina, San Diego, CA, USA).

### Positron emission tomography/computed tomography and contrast-enhanced CT

Positron emission tomography/computed tomography (PET/CT) was applied to identify the target lesions of distant metastasis, while contrast-enhanced CT was performed to assess the efficacy of ALK inhibitor-based targeted therapy in the patient.

## Results

A 57-year-old woman presented with a 10-year history of uterine myomas by physical examination without significant discomfort and noticed progressive enlargement of the mass during a follow-up in November 2020. She then received surgical resection in December 2020 at an outside institution, and the excised mass measured 7 × 5 × 4 cm. The postoperative pathology of the mass suggested uterine leiomyosarcoma (LMS) by IHC staining: SMA(+), AE1/AE3 (−), Vimentin (+), Desmin (partial+), CD117 (−), ER (partial+), S-100 (−), PR (partial+), CD10 (partial+), CyclinD1 (partial +), P53 (partial+), CD34 (−), and Ki-67(30%+). She was followed up with routine postoperative clinic visits every 3 months and was diagnosed with progressive disease (PD) by contrast-enhanced CT in February 2021.

**Figure 1:**
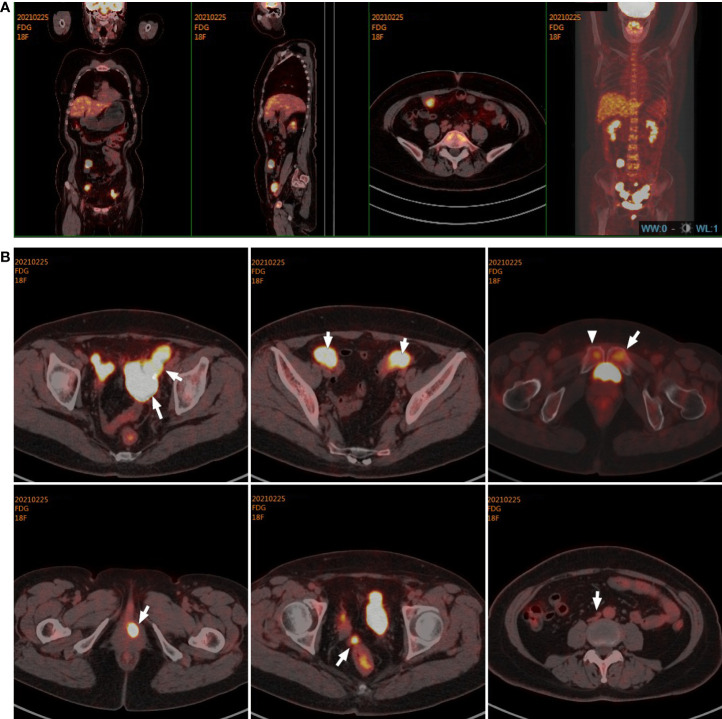
Target lesions of the patient with uterine IMT by PET/CT scans. **(A, B)** PET/CT scans indicated multiple metastases with increased ^18^F-fluoro-2-deoxyglucose (^18^F-FDG) uptake in the vaginal stump, left-side vulva, abdominal pelvic cavity, mesentery, and iliac paravascular and retroperitoneal lymph nodes in the abdomen and pelvic cavity. IMT, inflammatory myofibroblastic tumor.

**Figure 2:**
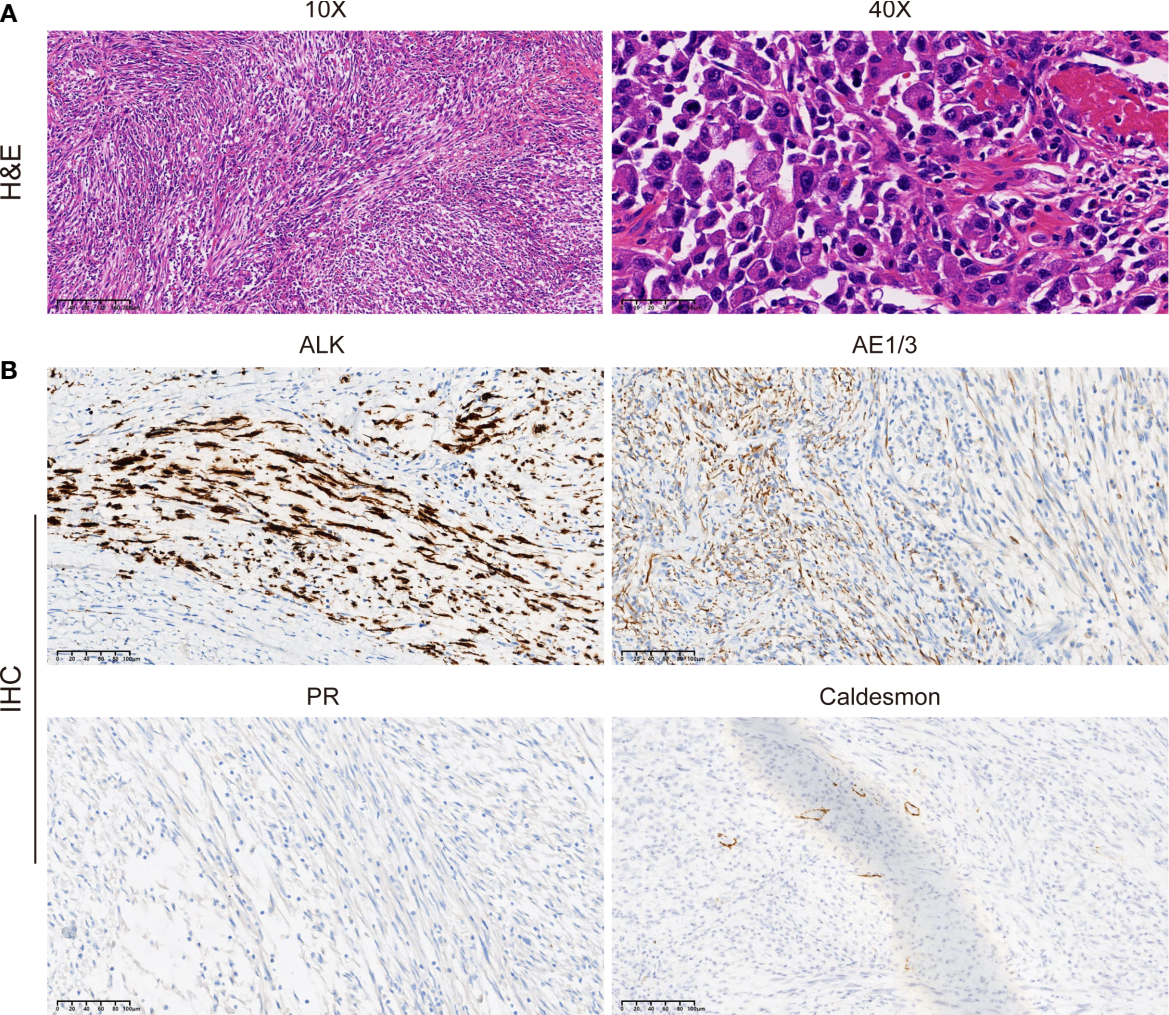
Morphologic features and IHC staining of the uterine IMT samples. Representative pictures of H&E staining **(A)** and IHC analysis **(B)** of ALK, AE1/3, PR, and caldesmon expression levels in uterine IMT. Scale bars are illustrated in the pictures. IHC, immunohistochemistry; IMT, inflammatory myofibroblastic tumor; H&E, hematoxylin, and eosin.

**Figure 3:**
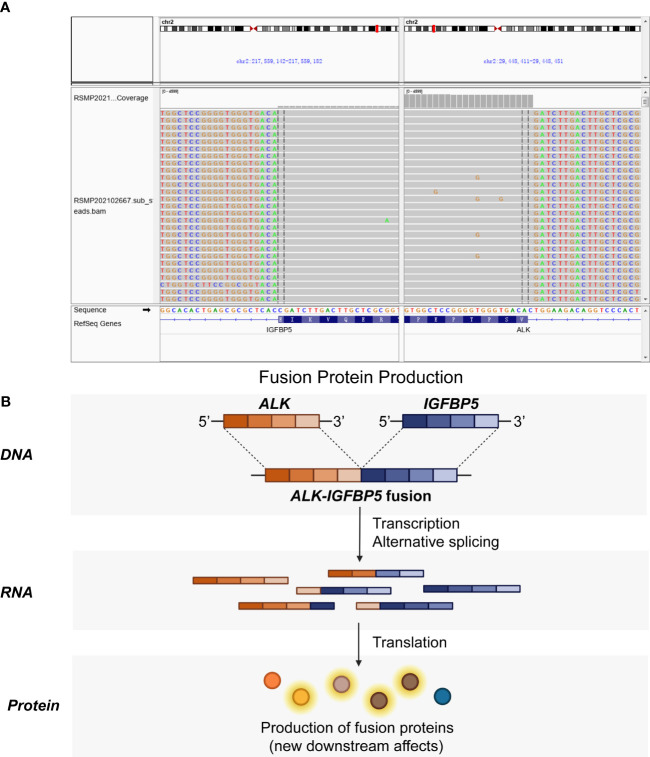
*ALK-IGFBP5* fusion within the uterine IMT samples. The *ALK-IGFBP5* gene fusion was identified by next-generation sequencing **(A)** and the working model **(B)** of the fusion. IMT, inflammatory myofibroblastic tumor.

Immediately afterward, the patient was evaluated at the Fudan University Shanghai Cancer Center for therapy recommendations. A whole-body PET/CT scan revealed multiple metastases with increased ^18^F-fluoro-2-deoxyglucose (^18^F-FDG) uptake in the vaginal stump, left-side vulva, abdominal pelvic cavity, and mesentery and iliac paravascular and retroperitoneal lymph nodes (**Figures 1A, B**). We, therefore, performed pathology consultation on the surgical resection specimens, and H&E showed myofibroblastic and fibroblastic spindle cells accompanied by the infiltration of plasma cells, lymphocytes, and eosinophils within the tumor (**Figure 2A**). IHC showed strong positive ALK [D5F3 (+)] staining, along with AE1/AE3 (partial+), MyoD1(−), caldesmon (−), BCOR (1+), HMB45 (−), CD10 (−), and PR (−) (**Figure 2B**). NGS was performed to detect the *ALK* rearrangement, and the result showed that there was an *ALK-IGFBP5* fusion in the tumor (**Figures 3A, B**). As we all know, approximately 50% of uterine IMTs are ALK-positive with *ALK* gene fusion (5). We, therefore, revised the diagnosis as uterine IMT according to diffuse ALK expression, *ALK-IGFBP5* gene fusion, and the morphologic features of the tumors by pathology consultation.

**Figure 4:**
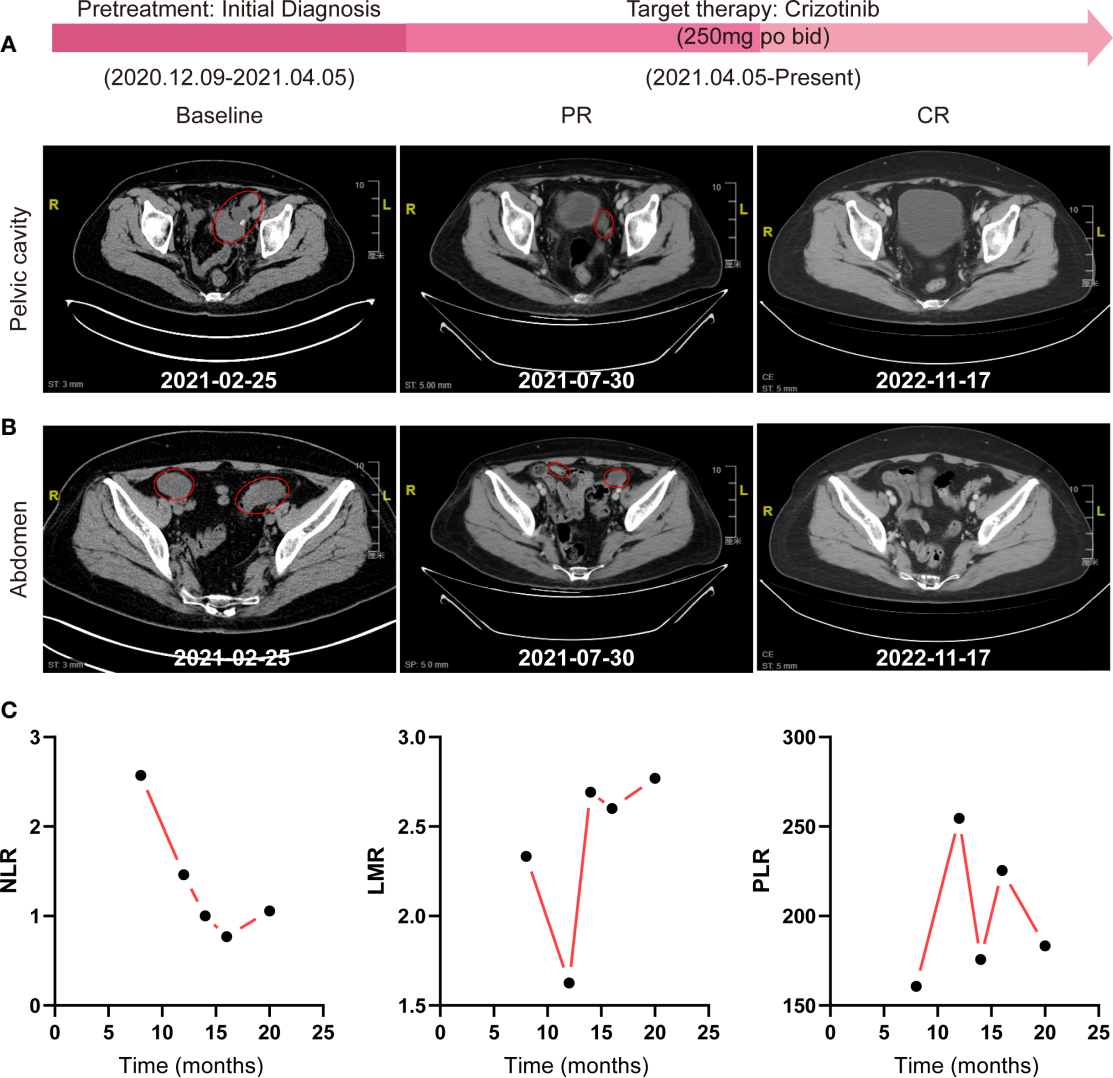
Effective evaluation by contrast-enhanced abdomen/pelvis CT scans and routine blood tests. **(A, B)** Radiographic assessments of target lesions by contrast-enhanced CT of the abdomen and pelvic cavity during the whole treatment process. **(C)** The change trends of NLR, LMR, and PLR were detected by routine blood tests. NLR, neutrophil-to-lymphocyte ratio; LMR, lymphocyte-to-monocyte ratio; PLR, platelet-to-lymphocyte ratio.

No further interventions were administered until recurrence after surgery, and the patient received an ALK inhibitor, crizotinib, at a dose of 250 mg bid orally for a 30-day cycle initiated on 25 April 2021. Tumor evaluations were performed with contrast-enhanced CT every two cycles thereafter. The patient achieved significant improvement in vaginal discharge, pelvic pressure sensation, and abdominal circumference after one cycle of crizotinib treatment. She tolerated the therapy well and presented with mild lower-extremity edema, which was controlled with spironolactone 40 mg tid for 5 days. After six cycles of treatment, the patient achieved a greater than 60% reduction in the sum of the longest diameter (SLD) of the target lesions according to RECIST 1.1 (**Figures 4A, B**), demonstrating a PR to therapy. She thereafter continued to receive crizotinib treatment with routine follow-ups to date. We observed the radiographic disappearance of all target lesions through contrast-enhanced CT scans of the abdomen and pelvis in November 2022, indicating a complete response (CR) with at least 18 months of progression-free survival (PFS) (**Figures 4A, B**).

Interestingly, we also observed a significant dynamic reduction of the neutrophil-to-lymphocyte ratio (NLR) by routine blood tests during the treatment process, while the lymphocyte-to-monocyte ratio (LMR) and platelet-to-lymphocyte ratio (PLR) showed no regularity (**Figure 4C**).

## Discussion

IMT is a group of mesenchymal tumors with a prevalence of 0.04% to 0.7% worldwide (1), which always develop in children or young adults but also can occur at any age. IMT usually begins in the lung but can also start in the larynx, stomach, liver, bladder, mesentery, and uterus (12), which makes uterine IMT a rare type of tumor. Accurate diagnostics is therefore particularly important for precision therapy because of the rarity of uterine IMT. In this case, we report a 57-year-old woman with recurrent uterine IMT who achieved a CR with at least 18 months of PFS after being administered conventional treatment with crizotinib without any adverse events. Our findings not only provide insight into the diagnosis and clinical therapy of uterine IMT but also offer experience in treatment monitoring and patient management.

In this case, the patient was initially diagnosed with uterine LMS by surgical pathology at an outside hospital. She then went to Fudan University Shanghai Cancer Center for therapy recommendations, and we updated the diagnosis to uterine IMT for subsequent treatment through pathology consultation. Accurate, specialized pathological diagnosis is fundamental to the clinical therapy of rare cancers. During the diagnostic process, uterine IMT is difficult to differentiate from LMS and uterine leiomyoma (UL) based on morphology alone (9). Due to the rarity of uterine IMT, most patients experience misdiagnosis or delayed diagnosis. From a morphological point of view, uterine IMT presented with myofibroblastic and fibroblastic spindle cells, accompanied by the infiltration of immune cells such as plasma cells, lymphocytes, and eosinophils (8). An immunohistochemical study is a gold standard for the diagnosis, as ALK positivity is specific to uterine IMT with a prevalence of more than 50% (13). ALK has now been acknowledged as a key driver in the progress of uterine IMT (6), as well as in NSCLC and other solid tumors (14). ALK positivity creates optimal opportunities for the generation of *ALK* gene fusion within a tumor, which has not been thus far reported in UL (15). Gene fusions drive the progression of 16.5% of cancers and serve as the sole driver in more than 1% of them (16, 17). Presently, there are nearly 30 different *ALK* fusion partners that have been identified across different types of tumors, while *FN1*, *DES*, *DCTN1*, *TNS1*, *IGFBP5*, *TIMP3*, *TNC*, *TPM3*, *THBS1*, and *SEC31* have been proven to exist in uterine IMT (7–10, 18). Among the 10 fusion partners, *IGFBP5* and *FN1* are both situated on the same chromosome as ALK, while *IGFBP5* and *THBS1* are the most frequent fusion types in uterine IMT (7).

In reality, different oncogenic *ALK* fusion partners always exhibit differential stability, leading to deregulated ALK activity along with differential sensitivity to ALK TKI. Wild-type ALK activates multiple signaling pathways such as PI3K-AKT, JAK-STAT, CRKL-C3G, MAPK, and MEKK2/3-MEK5-ERK5 pathways, to regulate cell growth and apoptosis (19). When fused with another gene, the protein conformation, stability localization, and function of ALK were changed, depending on the properties of the fusion partner (20). IGFBP5 has been reported to regulate cell survival, apoptosis, migration, and metastasis by IGF-dependent or IGF-independent mechanisms (21), and over-expression of IGFBP5 can confer resistance to PI3K inhibitors in breast cancer (22). Once fused with IGFBP5, ALK may function synergistically with IGFBP5 to trigger different signaling outputs that drive the initiation and progression of uterine IMT. However, the definite role and specific molecular mechanisms of how *ALK-IGFBP5* fusion drives uterine IMT progression still remain unclear and need further exploration.

Although recognized as an accomplice to carcinogenesis, ALK-positive tumors exhibit better overall survival (OS), providing possibilities for targeted therapy (23). Crizotinib, a first-generation TKI, was first approved to treat patients with ALK-positive NSCLC according to strong clinical response data (24). A previous study indicated that a patient with uterine IMT harboring *DCTN1-ALK* fusion suffered an ongoing PR after 6 months of therapy with crizotinib/Xalkori and pazopanib/Votrient (8). In another case, a patient with metastatic lung IMT with *TPM3-ALK* rearrangement obviously responded to ceritinib after progression on crizotinib (25). Further, a 66-year-old man with pulmonary IMT presenting with *GCC2-ALK* fusion was administered ensartinib therapy and achieved a PR (26), which provided a reference for patients who were crizotinib-resistant.

In addition, we also observed a significant decrease in NLR during the treatment process in addition to CT scans, which may provide a new reference index for efficacy evaluation. A high NLR has been acknowledged to be associated with unfavorable overall survival (OS) in several solid tumors (27), and to predict the prognosis of patients receiving immunotherapy (28, 29). However, no studies have validated the prognostic role of NLR in IMT-administered TKI target therapy. The detailed mechanisms underlying the correlation between high NLR and poor OS of patients with cancer may be due to high NLR, indicating an inflammatory and immunosuppressive status of patients (27). Further studies that involve more patients are required to determine whether NLR is an effective evaluation criterion. LMR and PLR also played a prognostic role in predicting the clinical outcome of patients with cancer (30, 31), although no correlation was observed in our case.

## Conclusion

We presented the case of a 57-year-old woman initially diagnosed with LMS, which was then corrected to uterine IMT based on morphology and *ALK-IGFBP5* fusion within the tumor. The patient was treated with crizotinib 250 mg initiated on 25 April 2021 and currently achieved CR with at least 18 months of PFS. This case may provide some insights for the precision diagnosis, target therapy, and post-intervention assessment of patients with uterine IMT harboring *ALK-IGFBP5* fusion.

## Data availability statement

The original contributions presented in the study are included in the article/supplementary material. Further inquiries can be directed to the corresponding author.

## Ethics statement

Written informed consent was obtained from the individual(s) for the publication of any potentially identifiable images or data included in this article.

## Author contributions

TZ, XZ, and ZL conceived the study, interpreted the data, and wrote the manuscript. XL, MR, YC, and JW collected the clinical data. XZ and ZL participated in data interpretation and discussions. All the authors read and approved the final manuscript.
